# Optimized upstream analytical workflow for single-nucleus transcriptomics in main metabolic tissues

**DOI:** 10.1093/lifemeta/loaf010

**Published:** 2025-04-02

**Authors:** Pengwei Dong, Shitong Ding, Guanlin Wang

**Affiliations:** Shanghai Key Laboratory of Metabolic Remodelling and Health, Institute of Metabolism and Integrative Biology, Centre for Evolutionary Biology, Fudan University, Shanghai 200438, China; Shanghai Key Laboratory of Metabolic Remodelling and Health, Institute of Metabolism and Integrative Biology, Centre for Evolutionary Biology, Fudan University, Shanghai 200438, China; Shanghai Key Laboratory of Metabolic Remodelling and Health, Institute of Metabolism and Integrative Biology, Centre for Evolutionary Biology, Fudan University, Shanghai 200438, China

Metabolic homeostasis is regulated by a network of organs and tissues, primarily involving adipose tissue, muscle, liver, and the hypothalamus, which act as central metabolic regulators. Cellular dysregulation within these tissues substantially associates with metabolic disorders, including obesity, type 2 diabetes, and non-alcoholic fatty liver disease (NAFLD) [1]. Understanding the molecular mechanisms governing metabolic control requires dedicated analysis of physiological and pathological cellular heterogeneity within these tissues. However, investigations at the single cell level to decipher the complexities of cellular mechanisms remain challenging due to the fragile nature of certain cell types and technical noise within these metabolically active tissues, resulting in limited studies compared to well-characterized atlases in immune cell populations [2].

Single-nucleus RNA sequencing (snRNA-seq) has emerged as an alternative approach to address these challenges [3]. By isolating nuclei rather than whole cells, snRNA-seq eliminates the necessity for extensive tissue dissociation, which is particularly advantageous for vulnerable cells or frozen samples. This approach enables high-resolution transcriptional profiling of metabolically active tissues and frozen tumor samples, revealing insights into cellular diversity and regulatory networks crucial for metabolic function. Despite these advantages, analyzing snRNA-seq data from metabolic tissues still presents unique difficulties. Adipose tissue is composed of large, fragile, lipid-rich adipocytes, along with diverse cell types, including preadipocytes, immune cells, and endothelial cells, all integral to energy storage and endocrine functions [4–6]. Similarly, muscle tissue comprises muscle fibers, satellite cells, and interstitial cells, which work together to regulate muscle metabolism [7], whereas liver tissue consists of hepatocytes, Kupffer cells, endothelial cells, and hepatic stellate cells, each with distinct gene expression profiles [8]. The heterogeneous cell populations complicate the transcriptomic analysis due to inherently lower mRNA content in nuclei compared to whole cells. Given these challenges, careful handling and analytical methods are essential for the interpretation of snRNA-seq datasets in metabolic tissues. To overcome the low abundance of transcripts in snRNA-seq datasets, the Bayesian-based normalization method *sctransform* has been developed to improve the identification of key cell types [9]. Another notable difficulty in single cell RNA sequencing (scRNA-seq) and snRNA-seq data analysis is the need for effective data integration and batch correction, especially when combining datasets from multiple samples, time points, or different labs for large consortia studies. Biological variations within and between samples can be masked by technical differences or batch effects. To address this challenge, several methods have been developed, including Seurat Canonical Correlation Analysis (CCA), Harmony, reciprocal principal component analysis (rPCA), single cell Variational Inference (scVI), and single cell Annotation Variational Inference (scANVI), etc. [10]. Each of these methods has unique strengths and limitations, making it essential to evaluate their performance on a case-by-case basis depending on the complexity and heterogeneity of the data. Thus, addressing these complexities of metabolically active tissues requires a systematic tailored upstream analytical workflow to extract meaningful biological insights from snRNA-seq datasets.

We introduce an optimized analytical workflow designed for snRNA-seq data from multiple metabolic tissues, including white adipose tissue, muscle, liver, and the hypothalamus, sourced from both human and murine models. It covers processing steps, including count matrix generation, ambient RNA removal, doublet identification, quality control (QC) of the expression matrix, normalization, data integration, and benchmarking, emphasizing the importance of ambient RNA and doublet removal for accurate cell type annotation. We have applied it to analyze 67 samples in 5 datasets (201,411 nuclei in total; Supplementary Fig. S1a) using 10× Genomics technologies (Supplementary Table S1) to show the performance.

The optimized and comprehensive upstream analysis workflow starts from raw FASTQ files (Fig. 1a). We used the standard Cell Ranger pipeline to generate an expression matrix, followed by applying CellBender [11] to remove ambient RNA contamination and scDblFinder for doublet removal [12]. The filtered matrix was then processed with Seurat for downstream analyses, including expression matrix QC (minimum/maximum expressed genes, minimum/maximum expression unique molecular identifiers (UMIs), and mitochondrial percentage), normalization, dimensional reduction, clustering, and annotation. After these preprocessing steps, 141,113 nuclei passed all the QC steps and were used in the downstream analyses (Supplementary Table S1). We incorporated multiple integration strategies, including Seurat CCA, Harmony, rPCA, scVI, and scANVI, followed by systematic benchmarking framework, the single cell integration benchmarking (scIB) [13], to help users decide the most appropriate integration method. This workflow ensures the reservation of high-quality data for subsequent cell-type classification and functional analysis, enhancing the interpretability and reliability of findings across heterogeneous metabolic tissues.

**Figure 1:**
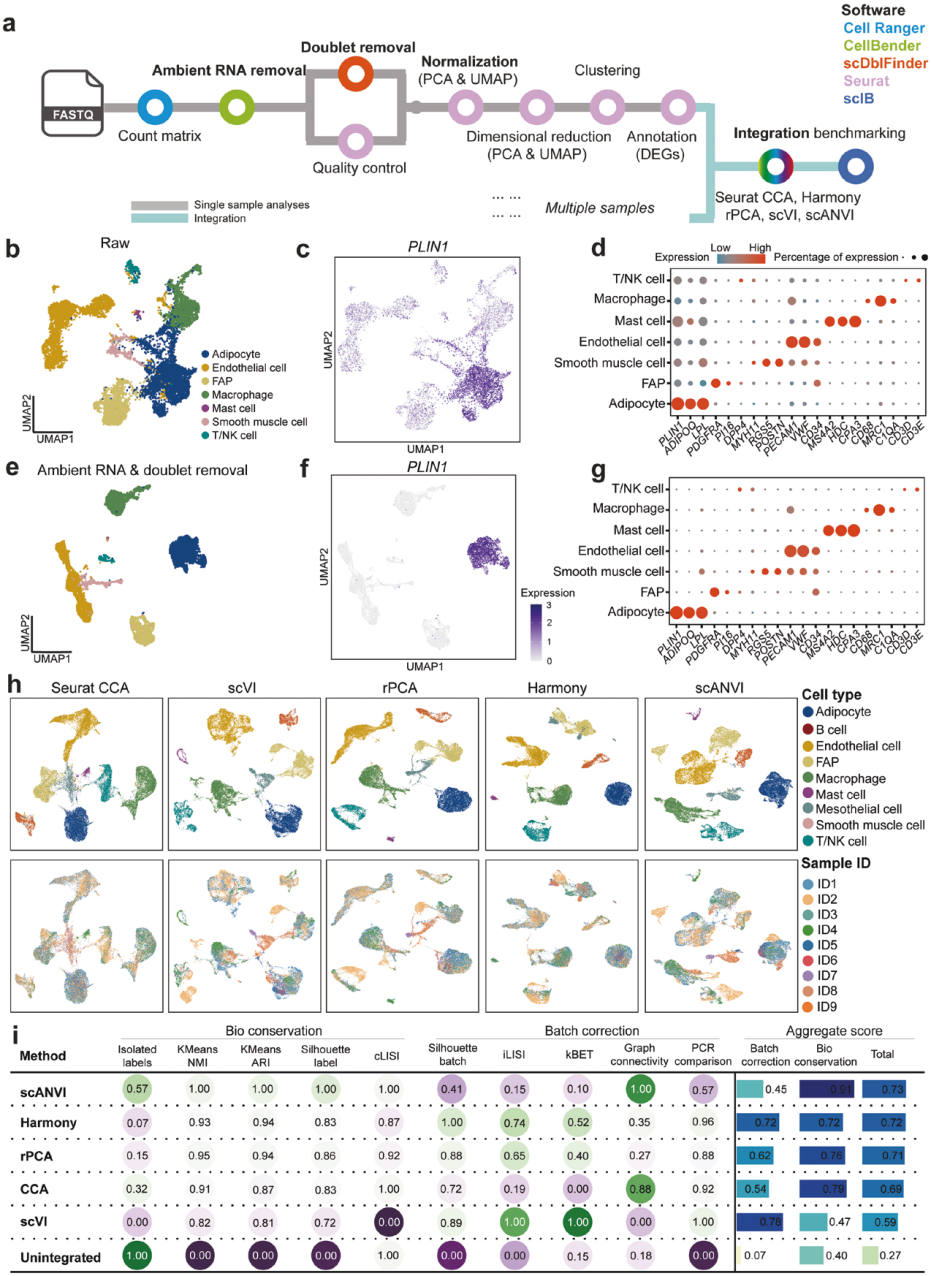
Overview and performance of the optimized snRNA-seq analysis workflow. (a) Schematic representation illustrates the optimized data processing workflow, starting with raw sequencing FASTQ files and proceeding to downstream integration analyses. Key steps include the generation of count matrices using Cell Ranger, removal of ambient RNA contamination with CellBender, doublet identification with scDblFinder, QC, normalization, dimension reduction (linear by PCA and non-linear by UMAP), annotation of cell types based on DEGs in Seurat, integration using methods such as Seurat CCA, Harmony, rPCA, scVI, and scANVI, and benchmarking using scIB. (b−d) Standard Seurat pipeline without ambient RNA and doublet removal. (b) UMAP projection of the raw data from one white adipose tissue sample, colored by annotated cell types: adipocytes, endothelial cells, FAP, macrophages, mast cells, smooth muscle cells, and T/NK cells. (c) Expression pattern of the adipocyte marker *PLIN1* in the raw dataset. (d) Dot plot depicts the expression levels of differentially expressed marker genes across cell types. Dot size represents the percentage of cells expressing each marker gene and color density reflects the average expression levels. (e−g) Standard Seurat pipeline after ambient RNA and doublet removal. (e) UMAP projections of cell types colored by cell type annotations after both ambient RNA and doublet removal. (f) Expression pattern of adipocyte marker *PLIN1* after ambient RNA and doublet removal. (g) Dot plot showing differentially expressed marker genes after both ambient RNA and doublet removal, indicating improved marker specificity and reduced background noise. (h) UMAP representations of the dataset after integration using five batch-effect correction methods: Seurat CCA, scVI, rPCA, Harmony, and scANVI. The first row shows UMAP representations colored by cell type, while the second row shows UMAP representations colored by sample ID. (i) The scIB framework is used for the quantitative assessment of batch-effect correction methods. Metrics are grouped into bio-conservation measures (isolated label performance, KMeans, normalized mutual information (NMI), etc.) and batch correction measures (integration local inverse Simpson’s Index (iLISI), *k*-nearest-neighbor batch effect test (kBET), etc.). Aggregate scores are calculated as a weighted mean (40:60) of batch correction and bio-conservation metrics.

Ambient RNA contamination and doublets commonly occur in the snRNA-seq library preparation during the isolation of the nuclei from individual cells, resulting in incorrect cell type annotation and consequently misleading biological interpretations. We compared the ambient RNA and doublet rates across various samples and tissues and found considerable variability across tissue types (Supplementary Fig. S1b and c), with higher ambient RNA rates in the liver (up to 90%) and white adipose tissue and higher doublet rates in white adipose tissue and the hypothalamus, highlighting the importance of removing ambient RNA contamination and doublets in snRNA-seq data in these tissues. CellBender was selected for ambient RNA removal after benchmarking against DecontX [14] and SoupX [15] across five datasets (Supplementary Fig. S1d). To evaluate the impact of ambient RNA and doublet removal, we compared cell type annotation results before and after applying the optimized pipeline from two independent automatic annotation methods, SingleR [16] and Azimuth [17]. The results demonstrate a more distinct separation of cell types after applying the optimized pipeline (Supplementary Fig. S2a and b). We further examined the expression of the widely used adipocyte marker gene *PLIN1*, which encodes Perilipin 1, associated with lipid droplet coating, in an illustrated example from white adipose tissue in Fig. 1b–g. *PLIN1* was diffusely expressed across nearly all cells prior to the removal of ambient RNA (Fig. 1b–d). Other adipocyte markers, including adiponectin (*ADIPOQ*) and lipoprotein lipase (*LPL*), demonstrated similar patterns (Supplementary Fig. S3a). Additionally, adipocytes and macrophages were poorly separated, and we identified doublets expressing both adipocyte marker genes (*ADIPOQ* and *LPL*) and macrophage marker gene (mannose receptor C-type 1 (*MRC1*)) or endothelial cell marker gene (von Willebrand factor (*VWF*)) (Supplementary Fig. S3a).

After the ambient RNA and doublet removal, *PLIN1* was exclusively expressed in the adipocyte cluster (Fig. 1e–g), reflecting improved cluster separation and enhanced marker gene specificity, as illustrated in uniform manifold approximation and projection (UMAP) plots, violin plots, and density plots (Supplementary Fig. S3a−d). Dot plots for the differentially expressed marker genes presented refined cell-type resolution, including adipocytes (*PLIN1*, *ADIPOQ*, and *LPL*), fibro/adipogenic progenitors (FAPs) (*PDGFRA*, *PI16*, and *DPP4*), endothelial cells (*PECAM1*, *VWF*, and *CD34*), macrophages (*CD68*, *MRC1*, and *C1QA*), mast cells (*MS4A2*, *HDC*, and *CPA3*), smooth muscle cells (*MYH11*, *RGS5*, and *POSTN*), and T cells (*CD3D* and *CD3E*) (Fig. 1g). We further examined the metabolic pathway enrichment and found that adipocytes exhibited notably higher enrichment for both fatty acid elongation and degradation pathways, consistent with their established role in lipid metabolism (Supplementary Fig. S4). This improvement also extended to other metabolic tissues we studied (Supplementary Fig. S5−7). We observed clearer cell-type clusters of the myofiber (*TTN*) in muscle (Supplementary Fig. S5), the hepatocyte population (*ASGR1*) in the liver (Supplementary Fig. S6), and the neuron (*Meg3*) in the hypothalamus (Supplementary Fig. S7) after ambient RNA and doublet removal. These steps significantly improve marker specificity and cell-type resolution across metabolic tissues, providing a robust basis for differentially expressed genes (DEGs) that are involved in the interpretation of cell-specific functions and metabolic homeostasis.

A key challenge in multi-sample snRNA-seq studies is the presence of batch effects, which can mask biological signals and introduce technical bias. To tackle this issue, we applied and benchmarked a selection of batch correction/integration methods, including Seurat CCA, Harmony, rPCA, scVI, and scANVI, across datasets to align cellular profiles across multiple samples and evaluate their performances based on their ability to preserve tissue-specific cell types and minimize batch-associated artifacts. We observed significant sample-specific clustering on UMAP embeddings, indicating technical variability rather than true biological variation in the illustrated adipose tissue dataset before batch correction (Supplementary Fig. S8a and b). After applying various methods, we observed substantial improvements with all integration methods, to varying degrees (Fig. 1h). We adopted the scIB weighted evaluation metrics, which have been benchmarked for evaluating the integration performance. In the illustrated adipose tissue dataset, we found that scANVI was better than other methods in conserving the unique biological transcriptional signatures (score = 0.91) of each cell type across samples with a moderate batch correction score (score = 0.45) (Fig. 1i), achieving the highest aggregated score for integration and clear cell-type separation (Fig. 1h). Harmony ranked the second-best integration method in this dataset with both relative high score in batch correction (score = 0.72) and biological signal conservation (score = 0.72) (Fig. 1i). Moreover, we found that Harmony consistently performed well for the liver (Supplementary Fig. S9), human muscle, and the hypothalamus [18]. Taken together, our benchmarking of integration methods highlights the need for careful method selection for processing multi-sample snRNA-seq data and the most appropriate method may vary depending on the specific data. Harmony has emerged as an effective method across tissues, making it the first choice for dataset integration.

Our study aimed to delineate a standardized upstream analytical workflow for snRNA-seq data in metabolically active tissues, including white adipose tissue, muscle, liver, and the hypothalamus, to achieve accurate cell-type clustering, annotation, and functionally relevant cellular insights by addressing technical challenges, including ambient RNA and doublet contamination, and rigorously benchmarking the integration of data from multiple samples. Our workflow enhances the resolution of cell clusters and ensures that subsequent analyses are based on high-quality, biologically meaningful data, facilitating the exploration of tissue-specific mechanisms underlying metabolic homeostasis and diseases.

We found substantial tissue-specific variability in ambient RNA and doublet rates. Liver and adipose tissue showed notably higher ambient RNA rates compared to other tissues. These variations likely arise from differences in tissue architecture, cell size, and nuclear RNA content, significantly affecting data quality and analytical approaches. Understanding these tissue-specific characteristics is crucial for optimizing preprocessing parameters and ensuring accurate biological interpretation. A major advancement in our work is the integration of established tools, specifically optimized for metabolic tissues in addressing ambient RNA contamination and doublet removal. With thousands of packages available in the single cell field, selecting appropriate tools can be challenging for biologists. Our workflow addresses this challenge by carefully benchmarking these tools, showing that CellBender and scDblFinder outperform other packages in metabolic tissue analyses. Ambient RNA contamination, originating from extracellular RNA that enters nuclear suspensions, can result in non-specific gene expression across different cell types, hence obscuring true biological signals. We showed that an ambient RNA removal step can effectively minimize this noise, allowing accurate identification of cell-type specific markers. *PLIN1*, a hallmark adipocyte marker gene, was shown to be exclusively expressed in adipocytes only after ambient RNA removal, as well as other marker gene expression in the muscle, liver, and the hypothalamus, confirming the necessity of this preprocessing step for accurate cell type annotation. This improved cell-type resolution (e.g. adipocytes) and identification of biologically relevant pathways are crucial for understanding metabolic homeostasis and disease states such as differences in the white adipose tissue in obesity and type 2 diabetes, where precise characterization of cellular subpopulations is essential for unraveling tissue remodeling and inflammation processes.

The integration of multi-sample datasets presented a key technical challenge that we addressed by benchmarking several batch correction methods, including Seurat CCA, Harmony, rPCA, scVI, and scANVI. Effective batch correction is crucial in multi-sample studies to reduce technical variability without compromising biologically meaningful cell-type differences, representing the most challenging analytical step in a large consortium/atlas [3, 10]. To address the difficulty of selecting the most appropriate integration method, we adopted the scIB framework, and its weighted evaluation metrics provide a systematic approach to method selection. We found that Harmony demonstrates strong performance in our presented datasets, whereas scVI also showed commendable results in previously published studies in the hypothalamus [19] and white adipose tissue [6]. Notably, users can adjust the parameters in the batch correction methods to enhance the compatibility of their datasets followed by scIB. For example, users can set varying *theta* values for different covariates in the Harmony method. Our benchmarking analysis emphasizes the necessity of choosing suitable integration methods for the complexity and composition of each tissue, reinforcing the importance of a standardized workflow to maintain biological integrity across samples.

In summary, our study underlines the important role of a standardized workflow in enhancing data quality and analytical reliability in snRNA-seq of metabolic tissue by combining rigorous preprocessing, cell-type clustering and annotation, and robust integration methods. By providing an open-access tutorial website (metabomicslab.github.io/snRNAseq-analysis-workflow/) and a Snakemake pipeline, we aim to make these analytical tools more accessible to the broader research community. This will broaden the application of single nucleus transcriptomics in metabolic biology to elucidate cellular heterogeneities and tissue-specific mechanisms and lay a strong foundation for future precision medicine targeting cell-specific functions.

## Limitations of the study

While our optimized workflow significantly enhances snRNA-seq data quality and analytical reliability, there are several limitations. First, the workflow requires basic computational resources and bioinformatics knowledge, which suggests that researchers should have a fundamental understanding of R or Python programming. To help with this, we have developed an open-access tutorial website and recommend cloud-based computing options to facilitate broader adoption. Second, tissue-specific variability in nuclear RNA content and cellular composition necessitates customized preprocessing parameters, as demonstrated by the distinct ambient RNA and doublet rates observed across different metabolic tissues. Although scIB provides an integrated evaluation framework, careful examination is still required to avoid overcorrection or loss of biological variability. Third, this workflow has been primarily optimized for snRNA-seq data, and its adaptability to other omics datasets, such as single cell assay for transposase-accessible chromatin sequencing (scATAC-seq), remains to be explored. Future improvements, including multi-omics integration and artificial intelligence-driven analytical tools, will be essential for expanding its applications across diverse research settings. Last but not least, we have not integrated downstream analyses into the current workflow, given the complexity and diversity of biological questions that need to be addressed.

## Supplementary Material

loaf010_suppl_Supplementary_Materials

loaf010_suppl_Supplementary_Tables

## Data Availability

All raw sequencing data are publicly available in GSE217677, GSE225700, GSE202379, PRJNA772047, and PRJNA771932, and all the re-analyzed Cell Ranger output files are available on Zenodo: zenodo.org/records/14725531.

## Code availability

All scripts used in the current study are available on GitHub: metabomicslab.github.io/snRNAseq-analysis-workflow/ and an integrated Snakemake pipeline (zenodo.org/records/14725531) for the upstream analyses.
