# Supplementary material for: Optimized upstream analytical workflow for single-nucleus transcriptomics in main metabolic tissues
Source: Life Metab. 2025 Apr 2;4(3):loaf010. doi: 10.1093/lifemeta/loaf010 (PMC12070799; doi:10.1093/lifemeta/loaf010)
Supplement: loaf010_suppl_Supplementary_Materials [file loaf010_suppl_supplementary_materials.docx]

**Supplementary Information**

**Methods**

**Datasets included in this study**

The raw sequencing data used in this study include mouse hypothalamus data (GSE217677) from Huang *et al.* [18], human white adipose tissue data (GSE225700) from Massier *et al.* [6], human liver data (GSE202379) from Gribben *et al.* [20], and both human (PRJNA772047) and mouse muscle data (PRJNA771932) from Scripture-Adams *et al.* [7].

**Single-nucleus transcriptomics data preprocessing**

Raw snRNA-seq reads in FASTQ format were downloaded from Gene Expression Omnibus (access code as above) and processed with Cell Ranger (version 7.1.0, 10x Genomics) for each sample and the reads were aligned to the GRCh38-2020-A genome for human samples and mm10-3.0.0 genome for mouse samples to generate unique molecular identifiers (UMIs) in the expression matrices.

**Ambient RNA removal**

CellBender (version 0.3.0) [11], DecontX (version 1.0.0) [14], and SoupX (version 1.6.2) [15] were employed to mitigate ambient RNA contamination in snRNA-seq data. The ‘raw_feature_bc_matrix’ was used as input for CellBender, which identifies and removes empty droplets. Before estimating contamination scores with SoupX and DecontX, low-quality cells were filtered out based on standard quality control metrics. For contamination estimation using DecontX, threshold values were set at 0.35 for white adipose tissue, while liver tissue, human and mouse muscle tissues, and mouse hypothalamic data were assigned a threshold of 0.4. Preliminary cell clustering was performed before computing contamination scores with the ‘autoEstCont’ function in SoupX to improve the accuracy of ambient RNA contamination estimation by identifying potential contamination sources more effectively.

**Quality control and doublet removal**

Individual samples were analysed using Seurat (version 5.1.0) [17] in R v4.3.1. Quality control filters were applied as follows: genes expressed in at least 3 cells, > 200 genes expressed per cell, > 500 UMIs per cell, and mitochondrial content < 5% for the hypothalamus and adipose tissue, and < 10% for muscle and liver samples. All samples were assessed for potential doublets using scDblFinder (version 1.16.0) [21], and nuclei identified as doublets were removed before downstream analyses.

**Normalization, clustering, and annotation**

Gene expression values were normalized using *sctransform* (v2) [9]. Principal component analysis (PCA) was carried out on variable features within the SCT assay, followed by Uniform Manifold Approximation and Projection (UMAP) analysis to reduce dimensionality to two dimensions using the ‘RunUMAP’ function with 25 to 40 principal components and a resolution range of 0.6 to 2. Cell types were manually annotated based on differentially expressed genes generated from the ‘FindAllMarkers’ function as well as the canonical marker genes from published literature (Supplementary Table S2).

**Automatic annotation**

Two independent computational methods, SingleR [16] and Azimuth [17], were employed for cell type annotation across five datasets. In SingleR, the BlueprintEncodeData reference was utilized for the adipose tissue and human muscle datasets, while the HumanPrimaryCellAtlasData was applied to the human liver dataset. For mouse muscle and hypothalamus datasets, MouseRNAseqData served as the reference. For Azimuth, the adiporef reference was used for adipose tissue, whereas the liver reference was applied for liver datasets.

**Biological insights assessment**

The scMetabolism [22] was applied to illustrate biological insights in adipose tissue data. The metabolic pathway enrichment scores were evaluated by using ‘sc.metabolism.Seurat’ function.

**Identification of differentially expressed genes**

Differential gene expression analysis was conducted using normalized counts stored in the data slot of the SCT assay. Prior to analysis, we first ran the ‘PrepSCTFindMarker’ function to set fixed values, followed by the ‘FindAllMarkers’ function with the Wilcoxon rank-sum test (assay = “RNA”, logfc.threshold = 0.1, min.pct = 0.1, only.pos = TRUE) to identify differentially expressed genes within each cell population, with an adjusted *P* value < 0.05.

**Data integration**

After quality control and preprocessing, mitochondrial genes, ribosomal protein-coding genes, and leukocyte antigen genes were excluded for downstream analyses. Batch effect was corrected using following integration tools: CCA, rPCA, and Harmony [23] implemented via ‘IntegrateLayers’ function in Seurat. We used all genes in scVI and scANVI integration (version 1.1.2) [24] and Seurat objects were converted to Anndata objects using scfetch (version 0.5.0) [25] in R with reticulate (1.37.0).

**Benchmarking evaluation matrices**

The benchmarking matrices, including Adjusted Rand Index (ARI) coefficients, LISI scores, and kBET rates, were used to assess integration quality across different donors. These metrics were calculated by the scib-metrics package (version 0.5.1) [13].

**Links for the websites in this study**

The open-access tutorial website:

<https://metabomicslab.github.io/snRNAseq-analysis-workflow/>

Zenodo:

https://zenodo.org/records/14725531

GitHub:

<https://metabomicslab.github.io/snRNAseq-analysis-workflow/>

**Supplementary Figures and Tables**

**
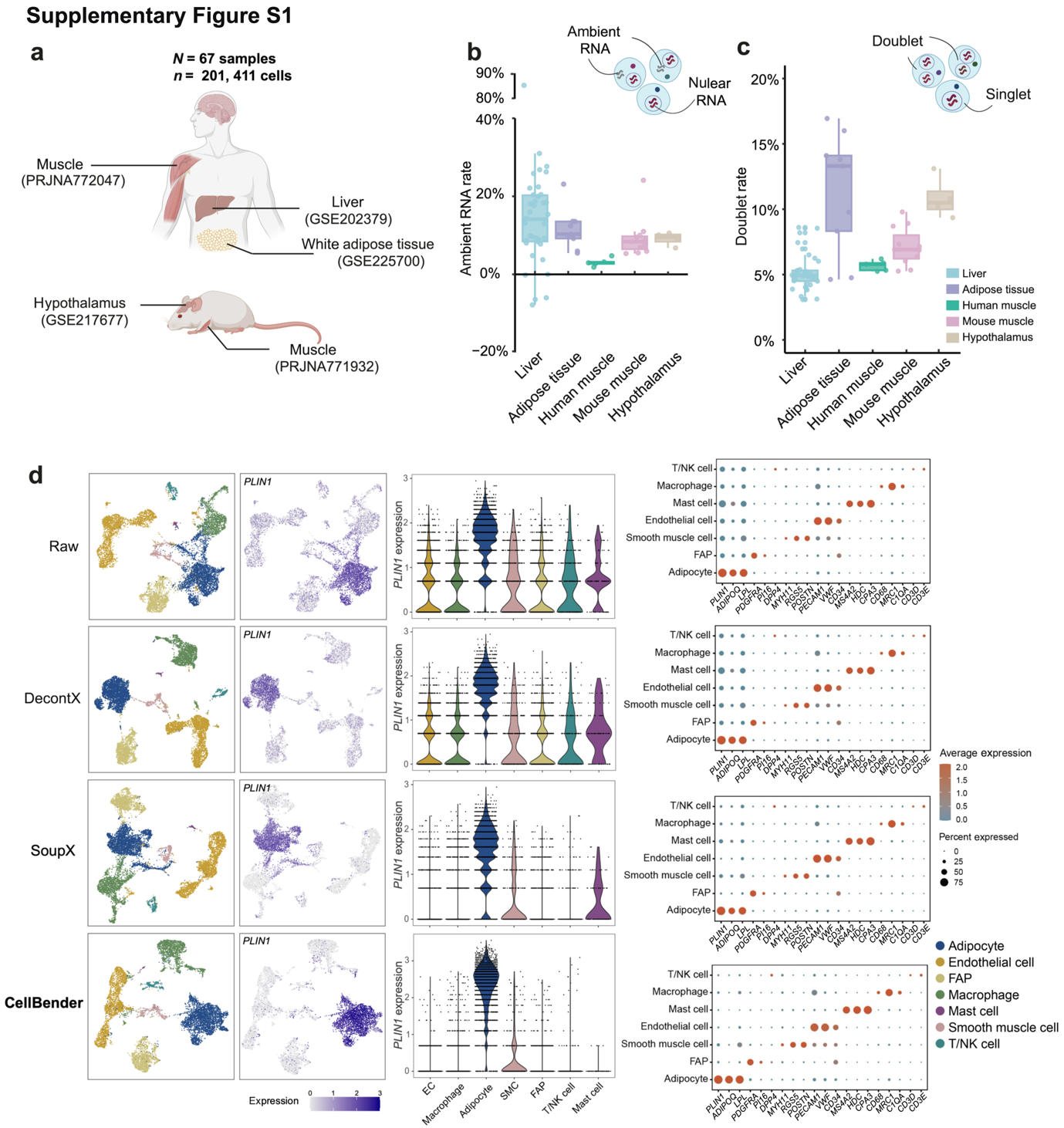
**

**Supplementary Figure S1** Overview of the datasets included in the study. (a) Overview of the datasets included in this study: white adipose tissue (GSE225700), muscle (PRJNA772047 and PRJNA771932), liver (GSE202379), and the hypothalamus (GSE217677). (b) Ambient RNA contamination across samples and tissues. Each dot represents a single sample. (c) Doublet rate across samples and tissues. Each dot represents a single sample. (d) Comparison of ambient RNA removal approaches in single-nucleus RNA-seq data from adipose tissue. Rows represent different processing methods—raw refers to standard Seurat workflow without ambient RNA, DecontX, SoupX, and CellBender (bottom). (Left) UMAP plots color by cell type. (Middle) Feature plots and violin plots showing expression levels of representative marker genes *PLIN1* across these cell types across different ambient RNA removal methods. (Right) Dot plots summarizing both the average expression and the fraction of cells expressing these genes in each cluster.


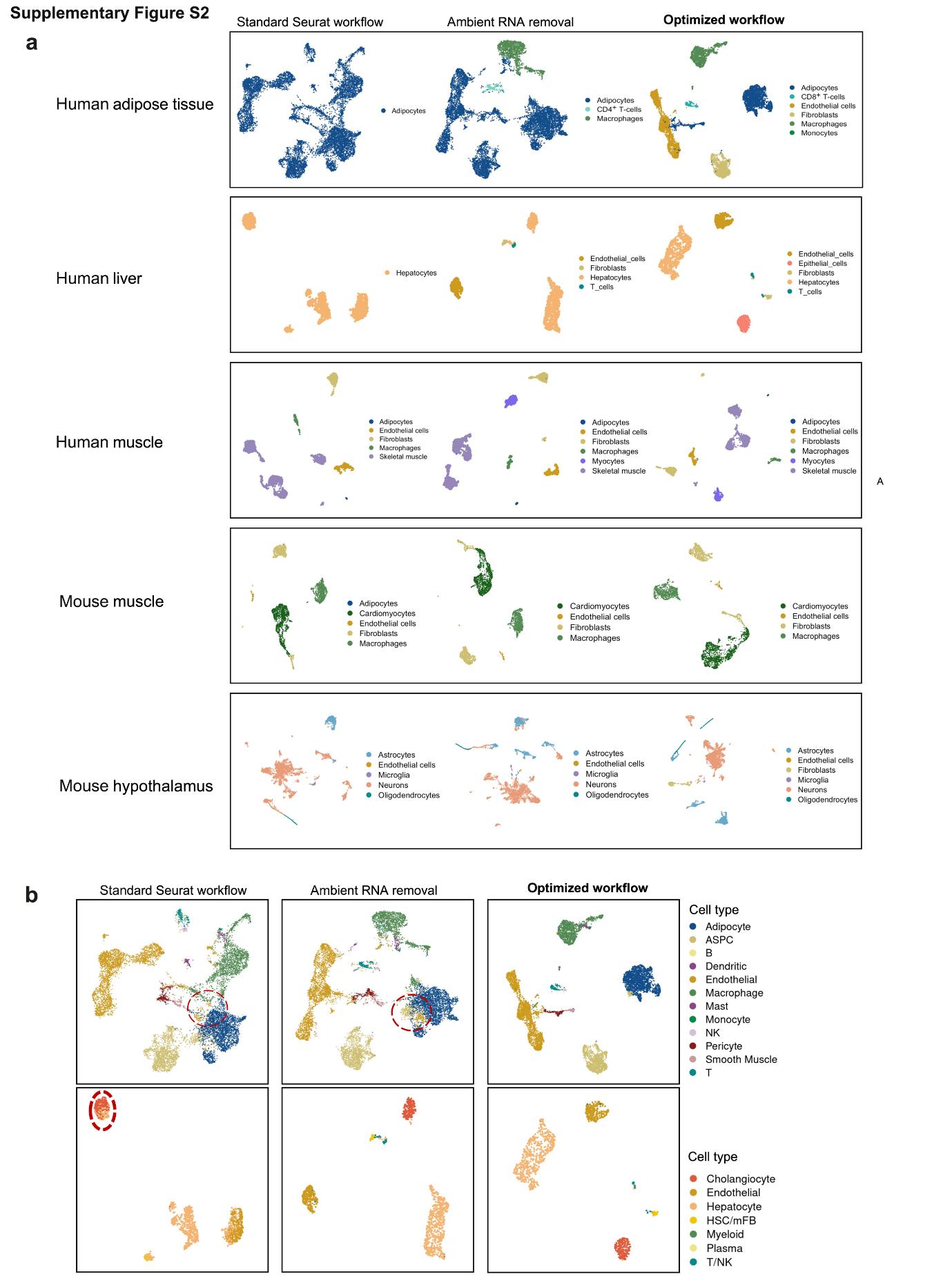


**Supplementary Figure S2** Cell type annotation performance of the optimized pipeline. (a) UMAP plots of cell type annotation by SingleR. UMAP plots illustrate the impact of ambient RNA removal and optimized pipeline on cell type annotation across five metabolic tissues. Columns represent the standard Seurat workflow (left), annotation after ambient RNA removal (middle), and annotation after the optimized pipeline (right). Each row corresponds to a distinct tissue: human adipose tissue, human liver, human muscle, mouse muscle, and mouse hypothalamus. Colors indicate different cell types, as shown in the legend. (b) UMAP plots of cell types annotated by Azimuth. (Upper row) UMAP plots illustrate the cell annotation results of adipose tissue datasets using the standard Seurat workflow (left), annotation after ambient RNA removal (middle), and annotation after the optimized pipeline (right). (Lower row) UMAP plots illustrate the cell annotation results of liver datasets using standard Seurat workflow (left), annotation after ambient RNA removal (middle), and annotation after the optimized pipeline (right), respectively. The red circles highlight mixed cell populations present without application of the optimized pipeline.


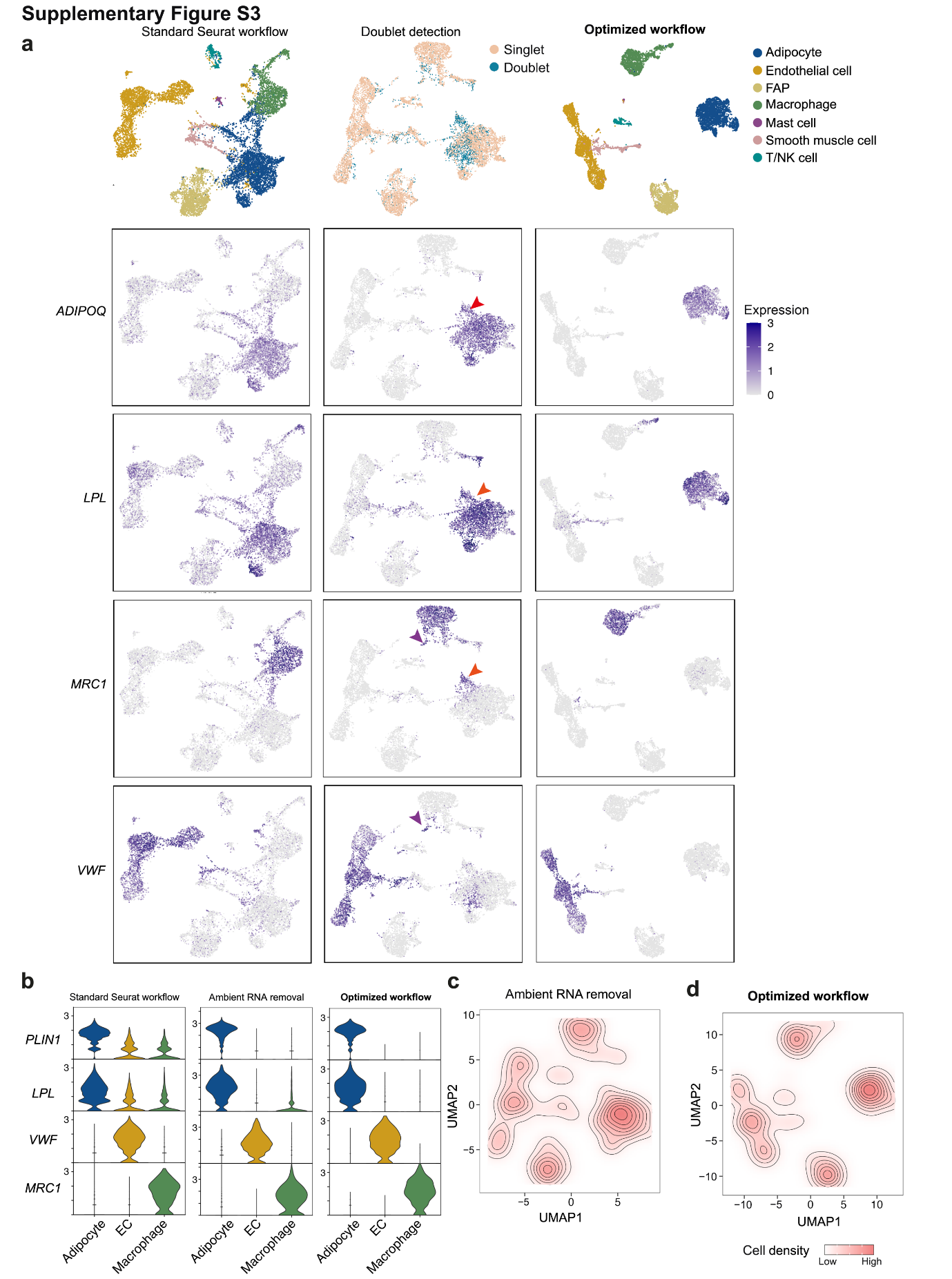


**Supplementary Figure S3** Effects of ambient RNA and doublet removal on cell type annotation and marker gene expression in white adipose tissue. (a) UMAP projections depicting cell type annotations and gene expression at different stages of data preprocessing. (Left) Raw data. (Middle) Singlets (light orange) and Doublets (dark green) identified after ambient RNA removal. (Right) UMAP after both ambient RNA and doublet removal. Expression patterns for marker genes, *ADIPOQ* and *LPL* (adipocytes), *MRC1* (macrophages), and *VWF* (endothelial cells), are displayed at each stage and red and purple arrows point to cells with multiple signature gene expression indicating high doublet possibility. (b) Violin plots comparing marker gene expression levels (*ADIPOQ*, *LPL*, *VWF*, and *MRC1*) across cell types at three stages: raw data, after ambient RNA removal, and after both ambient RNA and doublet removal. (c and d) Density plots showing cell density after ambient RNA removal (c) and after both ambient RNA and doublet removal (d). More accurate clustering is observed in the final dataset, reflecting improved data quality and cell-type resolution.

**
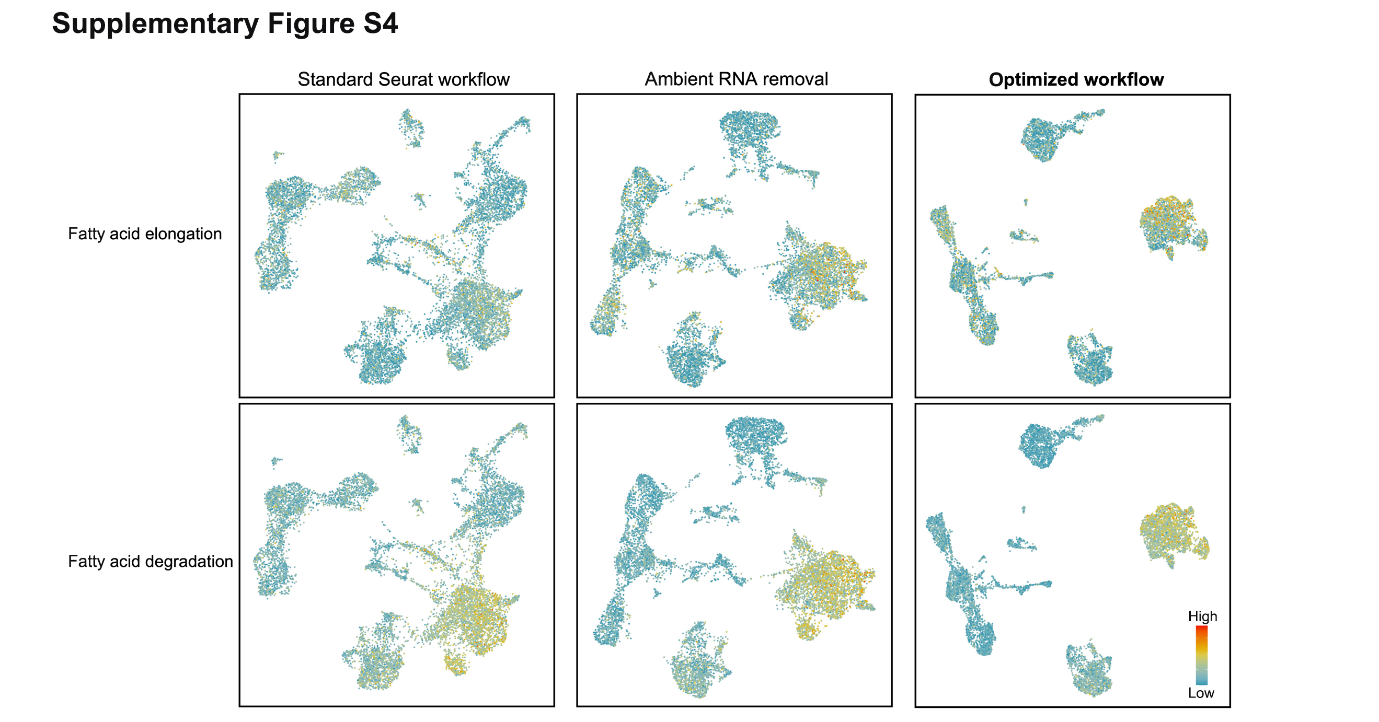
**

**Supplementary Figure S4** Enhanced biological insights derived from the optimized workflow, using adipose tissue as an example. Enrichment of fatty acid metabolism pathways was analyzed by scMetabolism. Standard Seurat workflow (left), annotation after ambient RNA removal (middle), and annotation after the optimized pipeline (right) are shown. The color scale indicates enrichment intensity (blue – low, yellow – middle, red – high).

**
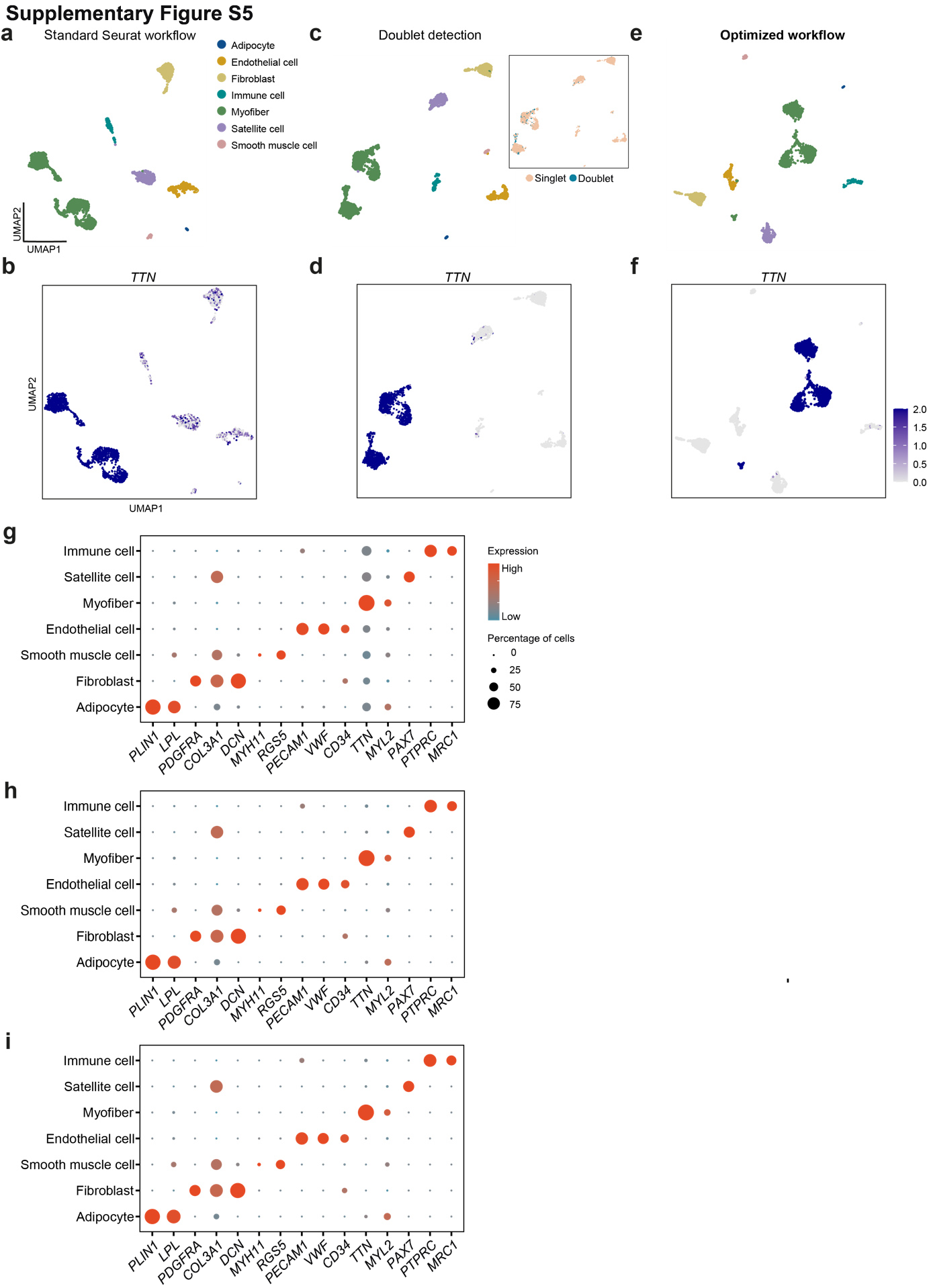
**

**Supplementary Figure S5** Impact of ambient RNA and doublet removal on muscle tissue cell type annotation. (a) UMAP projection of the raw data from a human muscle sample, coloured by annotated cell types: adipocytes, endothelial cells, fibroblasts, satellite cells, myofibers, smooth muscle cells, and immune cells. (b) Expression pattern of the myofiber marker *TTN* in the raw dataset. (c) UMAP projection illustrating cell types after ambient RNA removal without doublet removal and the distribution of singlets (light orange) and doublets (dark green) after ambient RNA removal. (d) Expression pattern of myofiber marker *TTN* after ambient RNA removal without doublet removal. (e) UMAP projection of cell types after both ambient RNA and doublet removal. (f) Expression pattern of myofiber marker *TTN* after ambient RNA and doublet removal. (g) Dot plot depicting the expression levels of differentially expressed marker genes across cell types. Dot size represents the percentage of cells expressing each marker and colour density reflects the average expression levels. (h) Dot plot showing marker gene expression after ambient RNA removal without doublet removal. (i) Dot plot showing differentially expressed marker genes after both ambient RNA and doublet removal, indicating improved marker specificity and reduced background noise.


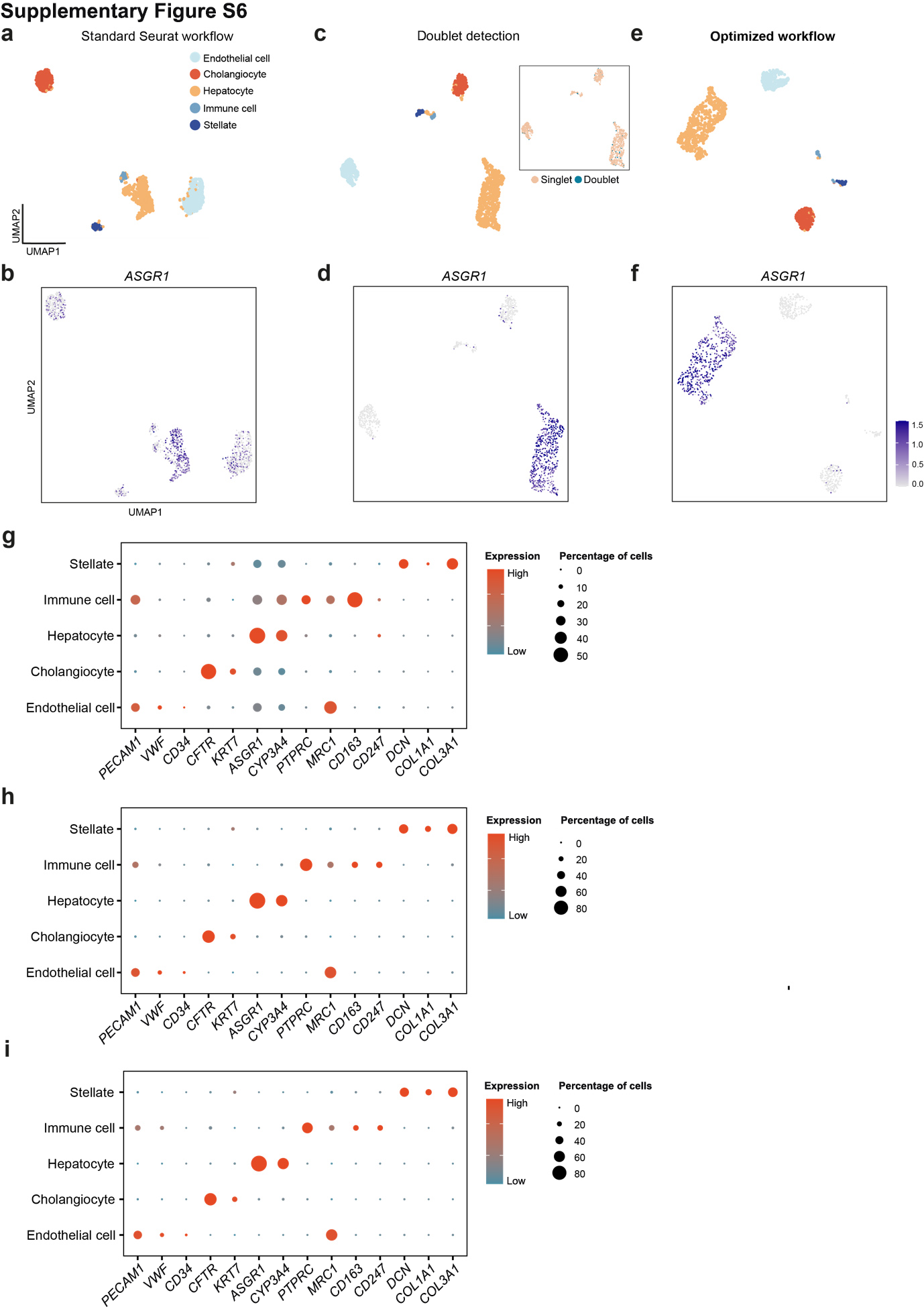


**Supplementary Figure S6** Impact of ambient RNA and doublet removal on liver tissue cell type annotation. (a) UMAP projection of raw data from a liver sample, colored by annotated cell types: stellate cells, endothelial cells, cholangiocytes, hepatocytes, and immune cells. (b) Expression of hepatocyte marker *ASGR1* on the raw data. (c) UMAP projection of cell types after ambient RNA removal without doublet removal and the distribution of singlets (light orange) and doublets (dark green) after ambient RNA removal. (d) Expression of hepatocyte marker *ASGR1* after ambient RNA removal without doublet removal. (e) UMAP projections of cell types colored by cell type annotations after both ambient RNA and doublet removal. (f) Expression of hepatocyte marker *ASGR1* after ambient RNA and doublet removal. (g) Dot plot showing the expression of differentially expressed marker genes across cell types. Dot size represents the percentage of cells expressing each marker and colour density reflects the average expression levels. (h) Dot plot showing marker gene expression after ambient RNA removal without doublet removal. (i) Dot plot showing the differentially expressed marker genes after both ambient RNA and doublet removal, indicating improved marker specificity and reduced background noise.

**
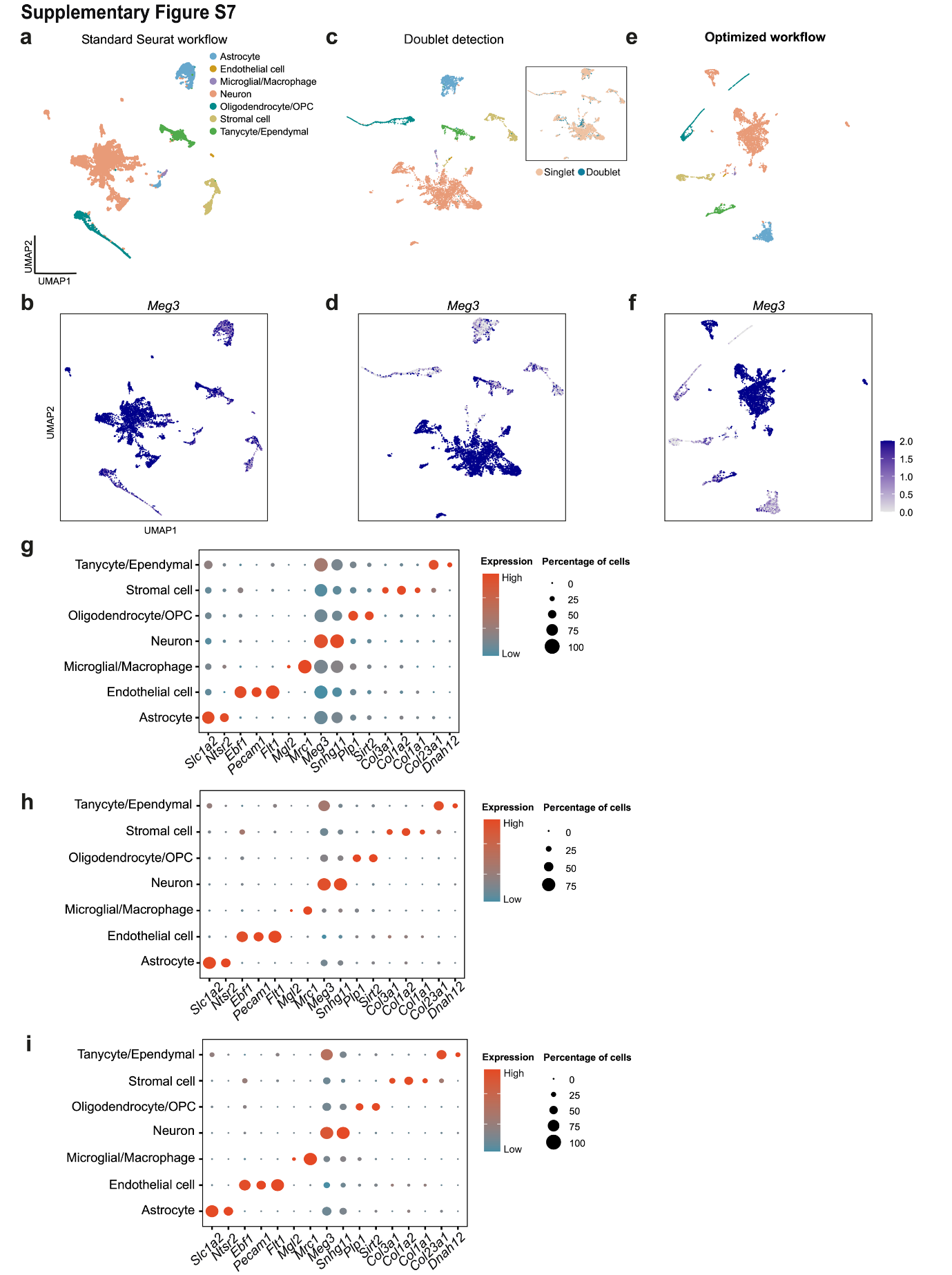
**

**Supplementary Figure S7** Impact of ambient RNA and doublet removal on the hypothalamus cell type annotation. (a) UMAP projection of raw data from a hypothalamus sample, colored by annotated cell types, astrocytes, endothelial cells, neurons, microglia/macrophages, oligodendrocyte/OPCs, stromal cells, and tanycyte/ependymals. (b) Expression of neuron marker *Meg3* on the raw data. (c) UMAP projection of cell types after ambient RNA removal without doublet removal and the distribution of singlets (light orange) and doublets (dark green) after ambient RNA removal. (d) Expression of neuron marker *Meg3* after ambient RNA removal without doublet removal. (e) UMAP projections of cell types colored by cell type annotations after both ambient RNA and doublet removal. (f) Expression of neuron marker *Meg3* after ambient RNA and doublet removal. (g) Dot plot showing the expression of differentially expressed marker genes across cell types. Dot size represents the percentage of cells expressing each marker and color density reflects the average expression levels. (h) Dot plot showing marker gene expression after ambient RNA removal without doublet removal. (i) Dot plot showing the differentially expressed marker genes after both ambient RNA and doublet removal, indicating improved marker specificity and reduced background noise.

**
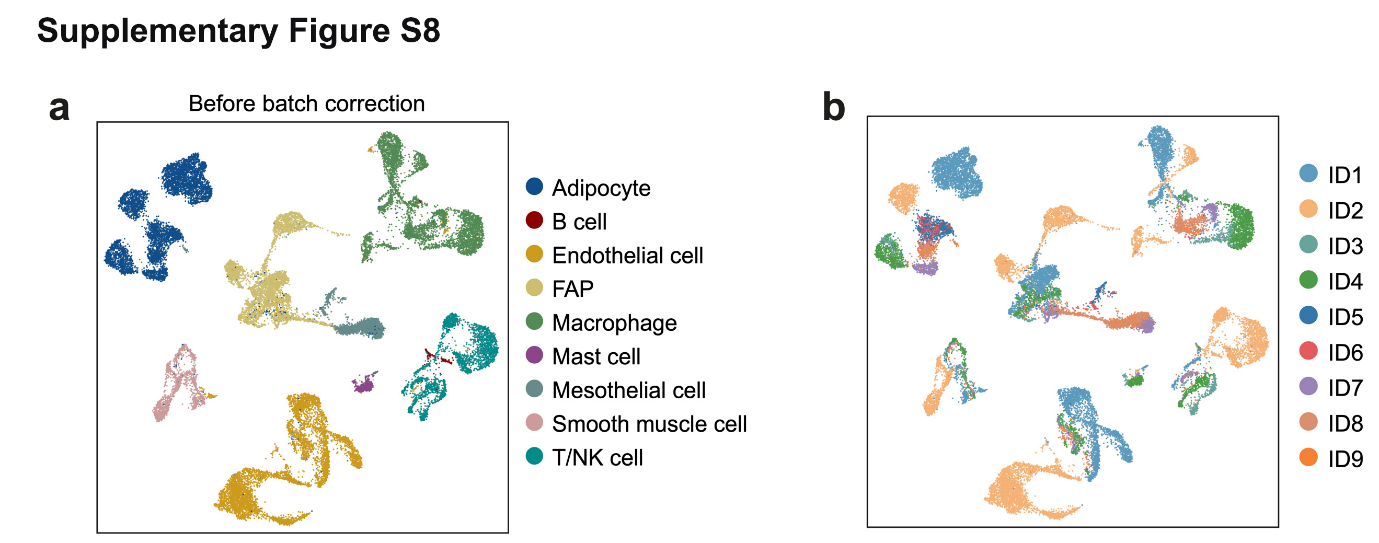
**

**Supplementary Figure S8** Sources of covariates in the illustrated adipose tissue dataset before batch correction. (a and b) UMAP projections on the merged dataset without batch correction, colored by the annotated cell types (a) and colored by donor IDs (ID1−ID9) (b), with batch effects showing distinct sample-specific clusters.

**
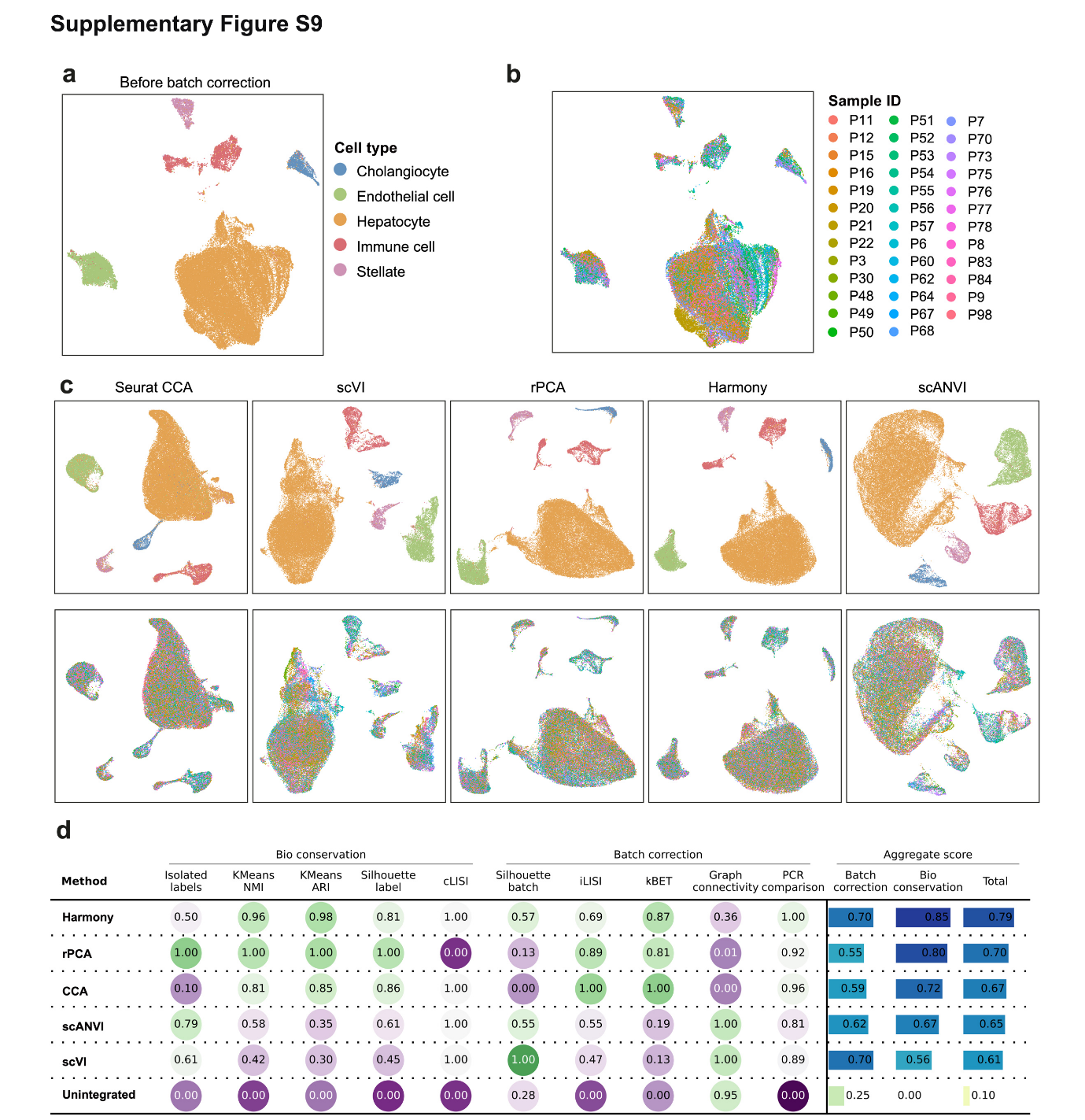
**

**Supplementary Figure S9** Benchmarking of integration methods in the liver atlas. (a and b) UMAP projections on the merged dataset without batch correction, colored by the annotated cell types (a) and colored by donor IDs (b), with batch effects showing distinct sample-specific clusters. (c) UMAP representations of the dataset after integration using five batch-effect correction methods: Seurat CCA, scVI, rPCA, Harmony, and scANVI. The first row shows UMAP representations colored by cell types, while the second row shows UMAP representations colored by donor ID. (d) The scIB framework used for the quantitative assessment of batch-effect correction methods. Metrics are grouped into bio-conservation measures (isolated label performance, KMeans NMI, etc.) and batch correction measures (iLISI, kBET, etc.). Aggregate scores are calculated as a weighted mean (40:60) of batch correction and bio-conservation metrics.

**Supplementary Table S1** Quality control matrices for individual samples of the datasets included in this study.

**Supplementary Table S2** List of canonical marker genes for annotating different cell types within the datasets of white adipose tissue, muscle, liver, and the hypothalamus.

**References**

[20] Gribben C, Galanakis V, Calderwood A *et al*. Acquisition of epithelial plasticity in human chronic liver disease. *Nature* 2024; **630**:166−73.

[21] Germain PL, Lun A, Garcia MC *et al*. Doublet identification in single-cell sequencing data using *scDblFinder*. *F1000Res* 2021: **10**:979.

[22] Wu Y, Yang S, Ma J *et al*. Spatiotemporal immune landscape of colorectal cancer liver metastasis at single-cell level. *Cancer discovery* 2022; **12**:134−53.

[23] Korsunsky I, Millard N, Fan J *et al*. Fast, sensitive and accurate integration of single-cell data with Harmony. *Nat Methods* 2019; **16**:1289−96.

[24] Luecken MD, Büttner M, Chaichoompu K *et al*. Benchmarking atlas-level data integration in single-cell genomics. *Nat Methods* 2022; **19**:41−50.

[25] Song Y, Gao J, Wang J. *scfetch*: an R package to access and format single-cell RNA sequencing datasets from public repositories. *bioRxiv* 2023. doi: 10.1101/2023.11.18.567507.
